# Scattered Leaves from a Physician’s Diary

**Published:** 1900-09

**Authors:** 


SCATTERED LEAVES FROM A PHYSICIAN'S DIARY.—A series of satirical sketches from real life, reflecting more or less upon the men who control it, by Albert Abrams, A. M., M. D. (Heidelberg), F. R. M. S., San Francisco, author of "The Antiseptic Club," etc.; pp. 60, with frontispiece; 50c. St. Louis, Mo.: Fortnightly Press Co., Publisher.

These sketches are very readable and cost only fifty cents, with the picture of the author thrown in. They consist of the following: "My First Patient," "A Scientific Courtship," "A Modern Esculapius," "A Mystery of the Latin Quarter," "The Imagery of Love," "The Euthanasia Club," "A Study of Light and Shadow," "A Martyr to His Profession," "A Patent Medicine," "Two Days in Spain," "The Professor of Bacteriology."

